# *Centella asiatica* as a Model Biomass for Sustainable Production of Biochemicals via Green Extraction and Purification Technologies: A Comprehensive Field-to-Market Review

**DOI:** 10.3390/molecules31030526

**Published:** 2026-02-02

**Authors:** Waqas Razzaq, Jean Baptiste Mazzitelli, Anne Sylvie Fabiano Tixier, Maryline Abert Vian

**Affiliations:** Avignon Universite, INRAE, UMR SQPOV, F-84000 Avignon, France; waqas.razzaq@univ-avignon.fr (W.R.); jean-baptiste.mazzitelli@univ-avignon.fr (J.B.M.); anne-sylvie.fabiano@univ-avignon.fr (A.S.F.T.)

**Keywords:** *Centella asiatica*, triterpenoid saponins, green extraction, sustainable cultivation, purification technologies, pharmaceuticals, nutraceuticals, cosmetics, circular bioeconomy, field-to-market approach

## Abstract

*Centella asiatica* has emerged as a strategic biomass for the sustainable production of high-value biochemicals at the interface of traditional medicine and modern biotechnology. This review consolidates the current knowledge on its phytochemical diversity, emphasizing triterpenoid saponins—asiaticoside, madecassoside, asiatic acid, and madecassic acid—as core bioactive molecules relevant to pharmaceutical, dermatological, nutraceutical, and functional-ingredient applications. Advances in green extraction technologies, including ultrasound-assisted, microwave-assisted, ohmic-heating, and supercritical CO_2_ systems, have demonstrated superior efficiency in recovering high-purity biochemicals while significantly reducing solvent use, energy demand, and environmental impact compared with conventional methods. Complementary analytical and standardization platforms, such as HPLC, UPLC, and GC–MS, enable rigorous quality control across the entire value chain, supporting the development of reproducible and regulatory-compliant biochemical extracts. From a biomass valorization and biorefinery perspective, *C. asiatica* offers multiple metabolite streams that align with circular economy and field-to-market sustainability principles. Key challenges remain, including agronomic variability, scaling up green extraction, and supply chain resilience. However, emerging solutions, such as Good Agricultural and Collection Practices (GACP) guided cultivation, plant tissue culture, metabolic engineering, and integrated biorefinery frameworks, show strong potential for establishing a reliable and environmentally responsible production system. Collectively, *C. asiatica* represents a model species for sustainable biochemical production, combining scientific efficacy with industrial, economic, and ecological relevance.

## 1. Introduction

*Centella asiatica* (L.) Urban (syn. *Hydrocotyle asiatica* L.), commonly referred to as Gotu Kola, is a perennial herbaceous plant belonging to the Apiaceae family [1,2]. The species is native to tropical and subtropical regions of South and Southeast Asia and parts of Africa. Outside its native range, it is widely cultivated and has been reported as locally naturalized in several regions, including Australia and some western Pacific islands; it is also grown in other tropical and subtropical areas, including parts of the Americas [2,3,4,5]. This species is the most extensively studied within its genus, owing to its rich ethnomedicinal heritage and broad applications in pharmacology and cosmetics [6,7,8]. *C. asiatica* holds a distinctive position at the confluence of traditional and contemporary medicine, being integral to various healing systems, including Ayurvedic, Chinese, Kampo, and African traditional practices [9,10,11]. In Ayurveda, it is mentioned in several classical texts, such as the Sushruta Samhita, under the names Mandookaparni or Brahmi, and is traditionally acknowledged as a “brain tonic” and “memory enhancer” because of its cognitive and neuroprotective properties [12,13]. It is prescribed for neurological disorders, including epilepsy, anxiety, senility, and premature aging [14]. Beyond its medicinal applications, the plant is consumed as a leafy vegetable and spice in various Asian cuisines [4,9]. Culinary applications vary by region: in Malaysia, it is included in salads such as “Ulam,” while in China and Thailand, it is prepared as a cooling beverage or herbal tea to aid digestion and urinary health [15]. Its affordability and availability, together with a generally favorable safety profile in traditional use, support its widespread use as both a food plant and a medicinal herb [16].

*Centella asiatica* is included in several authoritative pharmacopoeias and reference compendia, including the Indian Herbal Pharmacopoeia; the European Pharmacopoeia (including Ph. Eur. VI); the German Homeopathic Pharmacopoeia (GHP) and the German Pharmacopoeia (*Homöopathisches Arzneibuch*); and the Pharmacopoeia of the People’s Republic of China [8,17,18,19]. It is also documented in national references such as the British Herbal Pharmacopoeia, the Polish Pharmacopoeia (IX), the French Pharmacopeia, *La Farmacopea Italiana* (X), and Martindale, and its medicinal use is further documented in World Health Organization (WHO) monographs, which support standardized quality requirements [8,20,21]. *C. asiatica* is valued for its diverse bioactive compounds, especially triterpenoid saponosides like asiaticoside, madecassoside, asiatic acid, and madecassic acid, which have been reported to show wound-healing, neuroprotective, anti-inflammatory, and anti-aging effects [3]. This pharmacological potential has led to its use in functional foods, dietary supplements, pharmaceuticals, and cosmetics.

Alongside this market growth, the rising demand for natural ingredients has encouraged the adoption of green extraction and purification methods to increase bioactive recovery while reducing environmental impact [10]. From a biorefinery perspective, *C. asiatica* can be regarded as a high-value biomass for the production of bioactive compounds and value-added biochemical fractions, rather than only a traditional medicinal herb. Its triterpenoids, flavonoids, phenolic acids, sterols, and volatile terpenes correspond to the key biochemical fractions typically obtained from plant-based biorefinery systems, making it a suitable model for biomass valorization under green chemistry principles [3,22,23]. This perspective aligns with current green chemistry trends, positioning *C. asiatica* as a platform biomass for the sustainable production of high-value biochemicals using green extraction and purification technologies.

Beyond extraction performance, the diverse metabolite profile of *C. asiatica* supports its positioning within the emerging bioeconomy. Its phytochemicals can be fractionated into multiple biochemical streams, including pharmaceutical-grade triterpenoids, antioxidant phenolics, antimicrobial alkaloids, and nutraceutical compounds, supporting an integrated biomass valorization approach, where every component of the plant contributes to commercial and industrial value [22,24]. Due to its well-established characteristics, *C. asiatica* has gained significant commercial interest in dermatology and cosmetics [11,25]. Building on traditional use in wound care and skin inflammation, preclinical studies and selected human data have evaluated *C. asiatica* preparations for dermatological applications (e.g., scar-related and wound-related endpoints); however, outcomes remain formulation and indication-dependent and should be interpreted according to the level of evidence [11,26,27]. While the cosmetic sector remains a major driver of commercialization, the same biochemical constituents are also valuable in the pharmaceutical, nutraceutical, and functional food industries. Thus, *C. asiatica* should not be viewed solely as a cosmetic ingredient but as a multipurpose biochemical resource capable of feeding different bioproduct pipelines [24,28].

These use cases align with broader market drivers. The global shift towards natural and sustainable ingredients has created new opportunities for botanicals such as *C. asiatica* [29]. The bioactive ingredient market increasingly favors traceable, plant-based sources driven by health and environmental awareness. Consumers now prefer “clean-label” products that are safe, ethical, and minimally processed. This trend highlights the importance of transparency, sustainability, and simple product formulations supported by global standards that encourage responsible production and independent certification [30,31]. The global bioactive ingredients market, valued at USD 173.2 billion in 2022, is projected to reach USD 317.9 billion by 2030, with a CAGR of 7.9% [32]. Bio-based ingredients are increasingly preferred because of their lower environmental footprint and social responsibility, as they may reduce environmental burdens compared with some synthetic alternatives [33,34]. Reported geographical and agronomic variability can affect marker content, reinforcing the need for cultivation control and analytical standardization in industrial supply chains [35]. Regulatory frameworks, including ISO 14024 and the EU Ecolabel, further promote cruelty-free and resource-efficient production, enhancing consumer trust [30,31].

*Centella asiatica* is widely used in traditional medical systems and is now the subject of extensive modern phytochemical and process-focused research aimed at developing standardized extracts and industrially relevant bioactive fractions [36]. At the same time, increasing demand raises sustainability concerns across cultivation and processing. Traditional farming methods rely on chemical fertilizers and pesticides, which can cause water pollution and nutrient overload in water bodies [37]. In parallel, green extraction and purification technologies (e.g., ultrasound-assisted, microwave-assisted, and supercritical fluid extraction) can enhance process efficiency and help maintain the chemical integrity of bioactive constituents [38]. Where defined in monographs for specific preparations, minimum marker levels (e.g., ≥1% asiaticoside and ≥0.5% madecassoside) may be used for quality control; these thresholds depend on the cited monograph and extract type [22]. Overall, these developments support the recovery of a broader range of standardized fractions while reducing solvent and energy demand relative to conventional processing.

This review synthesizes current knowledge on *Centella asiatica* from a “field-to-market” perspective by integrating (i) cultivation and raw-material variability, (ii) phytochemical composition and marker-based standardization, and (iii) green extraction, solvent selection, and downstream purification strategies that enable production of triterpenoid and polyphenol-enriched fractions. The purpose is to critically evaluate how upstream agronomy and analytical control interact with extraction and purification choices to shape extract quality, scalability, and industrial applicability, while highlighting evidence level and key knowledge gaps relevant to biorefinery development.

The review is intended to benefit:

(i) researchers in natural products, pharmacognosy, food science, and green processing;
(ii) industrial stakeholders in cosmetics, nutraceuticals, and phytopharmaceutical manufacturing seeking scalable and compliant processing routes; and
(iii) quality and regulatory professionals involved in marker specification, standardization, and supply-chain traceability of *C. asiatica* materials.

### Literature Search Strategy

A structured literature search was conducted to synthesize evidence on green extraction technologies, solvent systems, downstream purification, and industrial applications of *Centella asiatica*. Searches were performed in PubMed, Scopus, Web of Science, ScienceDirect, MDPI, and Google Scholar using combinations of the following terms: *Centella asiatica*, triterpenoids, centellosides, green extraction, ultrasound-assisted extraction (UAE), microwave-assisted extraction (MAE), supercritical carbon dioxide extraction (SC-CO_2_), green solvents, natural deep eutectic solvents (NADES), purification, standardization, and biorefinery, along with application keywords (food, nutraceutical, cosmetic, pharmaceutical).

Studies published in English between 2000 and 2025 were considered. Eligible sources included experimental studies and review articles, alongside primary regulatory references where relevant (e.g., World Health Organization (WHO) monographs and European Pharmacopoeia. Publications were prioritized when they reported extraction parameters, solvent composition, quantified yields (with stated basis), phytochemical profiles, or defined purification/standardization workflows.

Search yield and screening: The search identified 500–530 records; after removal of duplicates (317), 289 titles were screened. Of these, 186 full-text articles were assessed for eligibility, and 146 publications were included in the final synthesis.

Publication trend: Across 2000–2025, publication output showed a clear increase, with the strongest growth after 2015–2025, driven mainly by studies on UAE/MAE process intensification, ethanol–water optimization, and the emergence of NADES and SC-CO_2_-based extraction and standardization approaches for triterpenoid-rich fractions.

## 2. Botany, Agronomy, and Cultivation of *C. asiatica*

**Figure 1:**
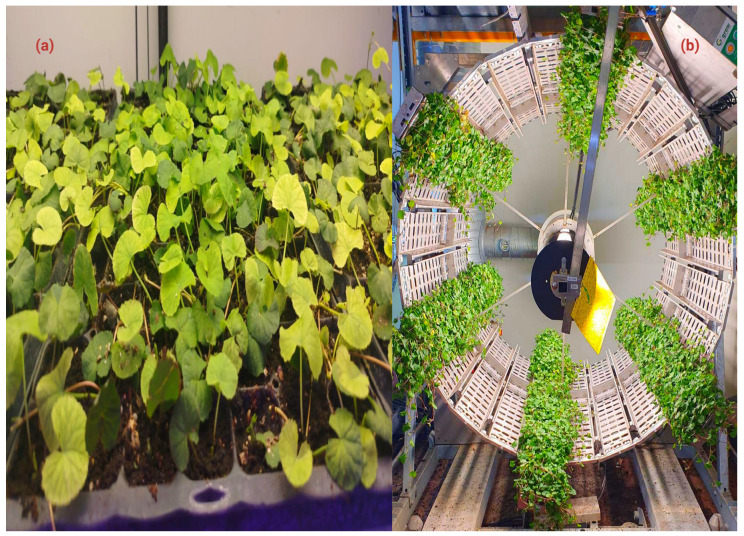
(**a**) *C. asiatica seedlings* during early-stage growth and (**b**) vertical farming cultivation under Controlled Environment Agriculture (CEA) at Futura Gaïa. These systems enable precise control of abiotic parameters, which can reduce environmental variability and support more uniform biomass production; the degree of phytochemical consistency depends on genotype and operating conditions (Images courtesy of Futura Gaïa, used with permission).

A clear understanding of the botanical traits and morphotypes of *C. asiatica* is important for optimizing cultivation and improving batch-to-batch consistency of marker content [3,5,39,40] (Figure 1a). *C. asiatica* is a prostrate, stoloniferous species that forms creeping mats with characteristic leaves. The species is diploid, with a chromosome number of 2n = 2x = 18 [40]. Considerable morphological variation exists among morphotypes, which has implications for their cultivation and industrial applications [41]. Studies comparing *C. asiatica* accessions report substantial genotype-dependent variation in centelloside accumulation, indicating that genotype selection is a key upstream lever for producing biomass with more consistent triterpenoid profiles. However, evidence for “high-centelloside” chemotypes is largely derived from field sampling, greenhouse cultivation, or laboratory-scale screening, and only a few studies provide directly comparable quantitative datasets across environments; therefore, reported advantages should be interpreted within the scale and conditions used in each study [5,28]. Similarly, reports that controlled-environment cultivation improves phytochemical consistency are mainly based on experimental or pilot-scale studies, and broader validation at an industrial scale remains limited. Current scientific evidence for pesticide-free, closed-loop cultivation systems specifically established for *Centella asiatica* is also limited. While hydroponic and in vitro culture systems can produce centellosides (often using sucrose, plant growth regulators, and elicitors such as methyl jasmonate), most studies focus on controlled propagation or metabolite stimulation rather than integrated closed-loop production systems [5]. In vitro propagation can generate genetically uniform plant material under laboratory conditions; however, the downstream therapeutic equivalence and performance of these propagated materials compared with conventionally grown plants are still insufficiently documented [35]. Genotypes exhibiting higher centelloside and phenolic levels are promising candidates for scale-oriented production, provided that performance and stability are confirmed under defined agronomic conditions. Together, these considerations support the selection of suitable genotypes and cultivation systems for the sustainable production of standardized biomass [42,43].

*C. asiatica* can be cultivated across a broad climatic range when adequate soil moisture is maintained, including tropical, subtropical, and some temperate production settings [3,4,9]. Asia remains the main cultivation hub for both medicinal and dietary purposes [41]. Although *C. asiatica* can be produced across a range of altitudes and cultivation systems, environmental conditions strongly influence biomass yield and phytochemical composition [4,9].

Accordingly, the agronomic performance and phytochemical quality of *C. asiatica* depend on climatic, soil, and management factors [28]. The plant grows best in warm, humid tropical or subtropical conditions (20–35 °C) and moist, marshy, or waterlogged sandy loam soils with a pH of 6.0–7.5 [40]. Annual yields range from 5 to 15 tons of fresh herb per hectare, depending on the genotype, environment, and cultivation management [5]. Soil composition and fertilizer type significantly influence the bioactive content [44]. For example, a soil–biocompost ratio of 25:75 combined with inorganic fertilizer increased total phenolic content by 33%, whereas a soil–biocompost–biochar ratio of 50:25:25 with organic fertilizer enhanced phenolic content by 30% and antioxidant activity by 16% [5]. These findings indicate that cultivation management is a major upstream determinant of marker accumulation and can therefore influence downstream extraction efficiency and standardization outcomes [22].

To meet the growing demand for high-quality, contaminant-free raw materials, modern cultivation increasingly focuses on sustainability and technological innovation [45]. Organic cultivation and controlled-environment agriculture (CEA) are increasingly explored to improve raw-material traceability and support more uniform production, with phytochemical outcomes depending on genotype and operating conditions [29,46,47]. Vertical farming and other controlled-environment approaches have been investigated as strategies to reduce environmental variability and improve batch-to-batch uniformity of biomass; reported effects on triterpenoid profiles depend on genotype, lighting regime, and nutrient management [47,48,49], as shown in (Figure 1b). CEA systems often rely on recirculating irrigation and may reduce pesticide inputs through physical exclusion and integrated pest management; however, pesticide-free operation should be stated only when documented for the specific production setting [49]. The increasing demand for traceable and sustainable raw materials from the cosmetic and pharmaceutical industries further supports the adoption of CEA-based cultivation [22,50]. Traditional production regions (e.g., Madagascar) remain important, while controlled cultivation may support more predictable supply and quality specifications for industrial value chains [51].

However, the successful positioning of *C. asiatica* as a reliable biochemical biomass depends on overcoming several production challenges. The plant is prone to fungal diseases, such as leaf spot and root rot, in excessively humid environments, which reduce yield [52]. Yield variability is another major issue influenced by genetic diversity, environmental fluctuations, and cultivation techniques [5]. Another challenge is the lack of raw-material standardization. Triterpenoid content can vary substantially across wild and cultivated materials from different regions [53]. Addressing these challenges is necessary for *C. asiatica* to function as a scalable and reliable biomass source for the industrial production of high-value biochemicals [5,22].

### Influence of Cultivation Conditions on Metabolite Production

Bioactive metabolite production in *C. asiatica* is influenced by environmental and agronomic conditions, as summarized below [5].

- Light: Light intensity and quality are major regulators of secondary metabolism [54]. Higher light levels can increase flavonoid, anthocyanin, and saponin synthesis, whereas low light can reduce asiaticoside and madecassoside accumulation. The red-to-blue light ratio is an important factor, with blue light often stimulating flavonoid biosynthesis [55]. Controlled-environment studies conducted in growth chambers and vertical farming systems have shown that 20% blue light at 200 μmol m^−2^ s^−1^ (typically under a 16 h photoperiod) promotes both growth and metabolite accumulation. In the cited study, triterpene glycosides and phenolic/flavonoid markers responded to the spectrum; however, AS/MS/AA/MA were not reported as absolute concentrations and were reported mainly as relative/comparative responses across treatments [56].
- Harvest time: Metabolite concentrations vary with the plant age. Plants harvested four months after planting contained the highest levels of madecassoside, asiaticoside, and their respective acids, coinciding with peak antioxidant activity [22].
- Nutrients: Nutrient availability, particularly phosphate, plays a key role. A moderate dose of 20 kg ha^−1^ phosphate maximizes triterpenoid, phenolic, and flavonoid synthesis in acidic soil [22]. In hydroponic systems, nutrient strength and electrical conductivity directly affect centelloside production, showing positive correlations with leaf number, leaf area, and total bioactive content [57].
- Microbial interactions: Plant growth-promoting rhizobacteria (PGPR), particularly the *Pseudomonas megaterium* strain HyangYak-01, significantly enhanced the synthesis of asiaticoside, madecassoside, asiatic acid, and madecassic acid. Metabolomic profiling has confirmed that PGPR treatment modifies metabolic pathways and increases the overall accumulation of secondary metabolites [37].

These cultivation factors collectively influence not only the chemical composition of *C. asiatica* but also its potential as a stable biomass source for industrial bioprocessing. Optimizing light, nutrients, harvest timing, and microbial interactions can help ensure consistent metabolite profiles and maximize triterpenoid and polyphenol production (Table 1). Such upstream control can facilitate downstream extraction and standardization; accordingly, cultivation optimization should be considered alongside green extraction and purification when designing biomass-to-biochemical workflows.

## 3. Chemical Constituents and Bioactive Metabolites

More than 130 metabolites have been reported across plant parts and analytical platforms, spanning triterpenoids (centelloids), flavonoids, phenolic acids, polyacetylenes, and phytosterols [53,70]. From a green chemistry perspective, this chemical diversity supports fractionation into multiple value streams using selective extraction and purification strategies [22,71,72].

### 3.1. Triterpenes (Primary Secondary Metabolites)

**Figure 2:**
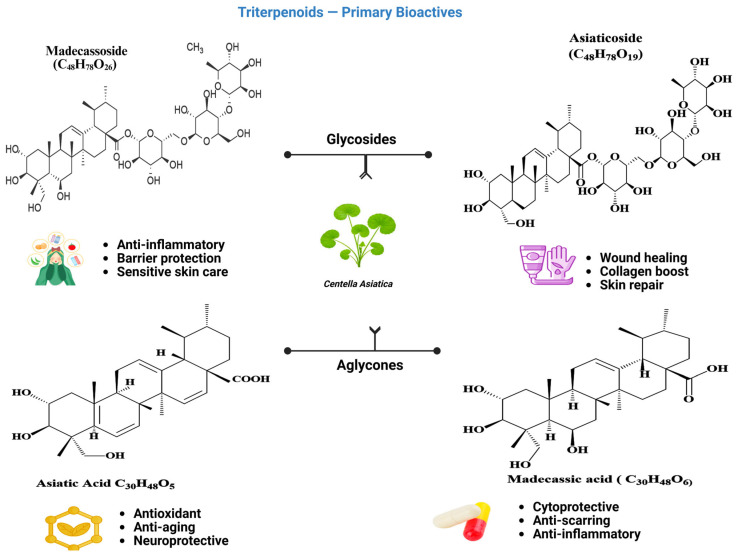
Primary centellosides of *C. asiatica*—asiaticoside, madecassoside, asiatic acid, and madecassic acid—and representative dermatological/pharmacological endpoints reported for these markers.

*C. asiatica* biosynthesizes pentacyclic triterpenoid saponins (centelloids), which constitute its main marker metabolites (Figure 2). Among them, asiaticoside, madecassoside, asiatic acid, and madecassic acid are widely used as marker triterpenoids and are among the most frequently reported bioactive constituents [15]. These triterpenoids are synthesized through the isoprenoid biosynthetic network involving the cytoplasmic mevalonate (MVA) pathway and plastidial methylerythritol phosphate (MEP) pathway. Subsequent oxidation and glycosylation processes, facilitated by cytochrome P450 monooxygenases (CYP450s) and glycosyltransferases (UGTs), respectively, result in the synthesis of asiaticoside, madecassoside, and their respective aglycones [17,73]. Structurally, these saponins contain hydrophobic aglycone backbones linked to hydrophilic sugar chains. Their biological activities largely depend on these structural features of the molecules. Asiaticoside and madecassoside are the principal glycosides, whereas asiatic acid and madecassic acid are the corresponding aglycones, which can exhibit distinct activity profiles compared with the glycosides depending on the model and endpoint [74]. These compounds mainly belong to the ursane-type triterpenes, although minor oleanane derivatives are also present [75]. Across many studies, glycosides are more abundant than aglycones, and both are frequently reported at higher levels in leaf tissues than in other organs, although distributions vary with genotype and cultivation conditions [76]. Triterpenoids represent the principal high-value biochemical stream targeted in most extraction and purification studies on *C. asiatica* for industrial standardization. Biosynthetic context can help interpret variability in marker profiles and guide selective enrichment strategies in biorefinery-oriented processing [22,77].

### 3.2. Other Bioactive Compounds

Depending on plant part, extraction conditions, and analytical workflow, approximately 50 non-triterpenoid constituents have been reported in targeted and untargeted studies (Table 2) [78,79]. Flavonoids constitute one of the largest classes, including quercetin, kaempferol, catechin, rutin, apigenin, naringin, castiliferol, and castilicetin [78,80]. Using spectrophotometric assays (AlCl_3_ for total flavonoids, expressed as mg QE/g; Folin–Ciocalteu for total phenolics, expressed as mg GAE/g), [81] reported indices of up to 345.17 ± 1.12 mg QE/g and 155.46 ± 0.52 mg GAE/g, respectively (basis as reported in the original study). These index-type totals are not directly comparable to concentrations of individual metabolites quantified by HPLC/LC–MS(/MS), which are typically reported in μg/g or mg/g depending on whether the matrix is dry plant material or dry extract [81]. In the same study, extract-level antioxidant activity correlated with flavonoid content (r^2^ = 0.894, *p* < 0.05), and the tested extracts showed antibacterial activity and thrombolytic and cytotoxic-type responses in vitro under the reported assay conditions; these findings should be interpreted as assay-dependent bioactivity rather than clinical evidence [81]. Some studies have reported additional low-abundance metabolites in targeted screens; however, when identification is based primarily on library matching without confirmation using authentic standards and MS fragmentation and NMR, these annotations should be considered putative [78,82]. Beyond their reported bioactivities, these constituents also represent extractable and recoverable biochemical streams that can be selectively valorized through the application of environmentally sustainable extraction and purification methodologies.

Recent LC–MS/MS metabolite profiling has identified 35 major compounds, primarily flavonoids, fatty acids, phenolics, and terpenes, with compositions that vary according to the genotype, geographical origin, environmental conditions, and extraction methods [79]. This chemical diversity underpins the broad interest in *C. asiatica* across pharmaceutical and cosmetic value chains [22,25]. This also reinforces the need for standardized cultivation, sampling, and analytical protocols to ensure reproducible marker profiles across studies and products. Furthermore, the extensive array of metabolites facilitates the establishment of multiproduct biorefinery models, wherein distinct classes of compounds are segregated into concurrent biochemical streams (e.g., triterpenoid, flavonoid, volatile, and nutritional fractions) [53]. Such approaches maximize the total biomass value and allow the production of multiple high-value biochemicals for pharmaceutical, nutraceutical, cosmetic, and biomaterial applications [23,85].

## 4. Green Extraction Technologies, Solvent Optimization, and Purification of Bioactive Triterpenoids from *C. asiatica*

**Figure 3:**
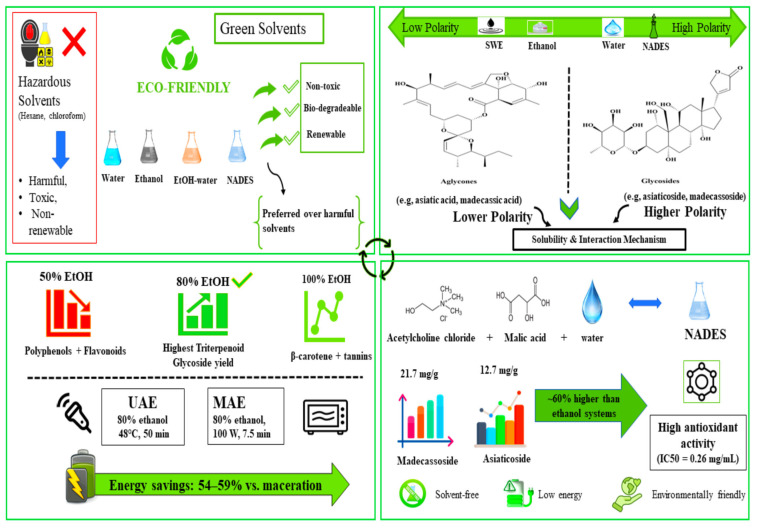
An overview of green solvent systems and the selective extraction mechanisms for bioactive compounds from *C. asiatica*.

Efficient extraction and downstream purification are central to producing standardized *Centella asiatica* fractions suitable for industrial use. In this context, the marker triterpenoids—asiaticoside, madecassoside, asiatic acid, and madecassic acid—are routinely targeted because they support quality control and represent major high-value constituents. Accordingly, recent work increasingly evaluates green and energy-efficient extraction approaches as alternatives to conventional solvent-based methods, aiming to improve selectivity and reproducibility while remaining compatible with scalable biorefinery workflows [86,87] (Figure 3). Throughout this section, reported yield differences are discussed in light of (i) solvent-driven selectivity (glycosides vs. aglycones) and (ii) cross-study methodological variability (extraction design, matrix, and reporting basis). Quantitative comparisons are interpreted with attention to analytical validation and inter-study comparability analytical method validation and comparability.

### 4.1. Conventional Extraction Methods

Conventional approaches—including maceration, reflux (heat-assisted extraction), Soxhlet extraction, and decoction—have historically served as baseline methods for isolating *C. asiatica* metabolites and remain widely used for preliminary screening and benchmarking newer technologies [36,76,88]. The main conventional extraction techniques commonly employed for *Centella asiatica* are summarized below. These methods differ mainly in temperature exposure, residence time, and solvent renewal, which influence selectivity and the risk of transformation.

- Maceration: Soaking plant material in a solvent at room temperature to dissolve metabolites.
- Reflux: Heating the plant material with a solvent under continuous boiling and condensation.
- Soxhlet Extraction: Continuous extraction in which the solvent repeatedly passes through the plant material.
- Decoction: Boiling plant material in water to extract water-soluble compounds.

However, their suitability for modern, large-scale, and sustainability-oriented processing is limited by (i) high solvent demand (often volatile organic solvents), (ii) long extraction times (hours to days), and (iii) limited selectivity, which can increase co-extraction of pigments, waxes, and other matrix components and thereby increasing the downstream purification burden [87]. In addition, when operated under prolonged heating (e.g., reflux/Soxhlet at or near solvent boiling temperature for several hours), these methods may promote transformation of labile constituents; for glycoside-rich targets, partial hydrolysis or shifts in the glycoside/aglycone balance have been reported under some conditions, potentially altering the extract composition relative to milder approaches [76]. For these reasons, conventional methods are treated here primarily as reference points, motivating the transition to green extraction technologies that reduce solvent and energy inputs and improve process controllability for standardized production [86,87].

### 4.2. Green and Eco-Friendly Extraction Approaches

Green extraction methods reflect a move toward more sustainable and environmentally responsible recovery of bioactive compounds from *C. asiatica.* They emphasize minimizing environmental impact and promoting sustainability during extraction [89]. The current focus is on methods that reduce solvent consumption and energy usage, decrease the number of processing steps, utilize safe and renewable natural materials, enhance extract yield and quality, and can be efficiently scaled up from laboratory to industrial applications [88,90].

Accordingly, assisted and intensified extraction technologies—such as MAE, ultrasonication, pulsed electric fields, high-pressure extraction, and supercritical fluid extraction—are increasingly favored over traditional methods because they offer higher extraction efficiency, greater selectivity, shorter processing times, and environmentally safer operations [76]. Specifically, industrial-scale applications of *C. asiatica* now include MAE, pressurized hot water flow-through extraction, supercritical CO_2_ (SC-CO_2_) extraction, ohmic heating-assisted extraction (OHAE), and ultrasonic-assisted extraction (UAE) [88].

The advancement of sustainable solvent systems is a key aspect of green extraction research. Supercritical CO_2_, for example, is non-toxic, non-flammable, and easily removed from the final extract, making it an eco-friendly alternative to conventional organic solvents [89]. Similarly, pressurized hot water extraction uses water at high temperature and pressure, reducing the reliance on organic solvents while preserving heat-sensitive bioactive compounds [89].

#### 4.2.1. Advanced Green Extraction Techniques

Several advanced green extraction technologies have been developed and optimized for recovering bioactive compounds from *C. asiatica*. Among these, microwave-assisted extraction (MAE) is frequently reported as an efficient approach because microwave heating can accelerate mass transfer, shorten processing time, and reduce solvent consumption relative to conventional methods, depending on solvent system and operating conditions [91]. In addition to solvent-based MAE, solvent-free microwave-based methods (often termed solvent-free microwave-assisted extraction, SFME) represent a distinct class of microwave processing in which no added organic solvent is used; extraction relies on the intrinsic moisture of fresh plant material (and, in some configurations, reduced pressure) to enable rapid processing [36]. For example, [36] reported processing 20 g of fresh *C. asiatica* for 15 min at 300 W under the stated vacuum/operating conditions, demonstrating a rapid solvent-free microwave workflow for obtaining a plant-derived extract/fraction under those conditions.

Ultrasound-assisted extraction (UAE) is another widely used approach for *C. asiatica* is another widely used approach for *C. asiatica*. UAE with ethanol–water mixtures (commonly ~70–80% ethanol, depending on study design and target analytes) has been reported to increase recovery of key triterpenoid glycosides such as asiaticoside and madecassoside while reducing extraction time and solvent use compared with conventional extraction [22]. The extent of improvement depends on matrix preparation, ultrasound conditions, and the reporting basis used in each study [53].

Supercritical fluid extraction (SFE; SC-CO_2_) is primarily used to recover lipophilic to moderately polar fractions from *C. asiatica*, such as volatile/terpenoid components and other non-polar constituents; when polar modifiers (e.g., ethanol) are added, SC-CO_2_ can also recover triterpenoid aglycones (asiatic acid, madecassic acid) and, to a lesser extent, more polar triterpenoid derivatives, depending on operating conditions and co-solvent level [22]. Because extraction is performed at relatively low temperatures under pressure, SFE can help limit thermal stress compared with prolonged reflux methods, and CO_2_ is readily removed from the final fraction after depressurization [22]. By contrast, enzyme-assisted extraction (EAE) targets matrix disruption rather than solvent substitution: cellulases/pectinases (and related enzyme cocktails) hydrolyze cell-wall polysaccharides, which can enhance the recovery of polar metabolites, notably triterpenoid glycosides (asiaticoside, madecassoside) and polyphenol-rich fractions, using milder aqueous or hydroalcoholic systems and reduced extraction severity [22,92]. Novel processing approaches, such as nanopowder production, offer additional sustainability benefits. Nanopowders of *C. asiatica* produced using a planetary ball mill showed ~50% higher asiatic acid recovery in water extraction than micropowders, offering a clean, eco-friendly, and cost-effective extraction process [93]. This approach eliminates the need for organic solvents and improves extraction efficiency.

#### 4.2.2. Hybrid and Combination Extraction Approaches

Combining green unit operations has emerged as a strategy to improve efficiency and tailor selectivity for *C. asiatica* extracts [53,76,94]. These approaches aim to exploit complementary mechanisms (e.g., cavitation-assisted matrix disruption followed by rapid microwave heating) to increase mass transfer and reduce processing time relative to single-step protocols. One frequently reported hybrid approach involves combining ultrasound-assisted extraction with microwave-assisted extraction (UAE-MAE). Research has demonstrated that the UAE-MAE extraction method with absolute ethanol solvent and *C. asiatica* particles < 355 μm was optimal for preparing *C. asiatica* extract in both lab-scale and scale-up experiments [88]. This combination method has been shown to significantly enhance extraction efficiency, with studies reporting that ultrasound pre-treatment followed by microwave-assisted extraction was investigated for the first time, specifically for *C. asiatica* compound extraction [95].

Natural deep eutectic solvents (NADES) can also be paired with assisted extraction to improve recovery of polar bioactives while avoiding conventional volatile organic solvents; Centella-specific examples include NADES-assisted extraction configurations reported in the cited studies [95]. Advanced hybrid strategies have demonstrated even greater complexity and efficiency. Hybrid extraction strategies, such as MAE-EAE (combining rapid thermal diffusion with enzyme specificity) and SFE-PLE (targeting diverse phytochemical phases), demonstrate synergistic benefits, with these integrated approaches maximizing yield, preserving compound integrity, and exemplifying the fusion of efficiency, sustainability, and phytochemical fidelity in next-generation *C. asiatica* extraction methods [22].

The industrial relevance of hybrid extraction is supported by reports that combining unit operations can improve efficiency and facilitate scale-up. For example, supercritical CO_2_ extraction coupled with MAE pre-treatment has been used to increase extraction yields, illustrating the potential of integrated workflows for industrial process design. Such integrations can reduce processing time and, in some cases, solvent and energy demand while maintaining or improving target-compound recovery, although outcomes depend on equipment configuration, feed matrix, and the target compound class [88,90].

#### 4.2.3. Sustainable Benefits and Environmental Impacts

Green extraction approaches can reduce solvent demand and processing energy relative to conventional maceration/reflux; however, sustainability performance is method- and context-dependent and should be interpreted using the reported operating conditions and normalization basis (e.g., per batch, per kg biomass processed, or per unit marker yield). For *C. asiatica*, [96] reported lower calculated energy demand for MAE and UAE than for maceration (≈59% and ≈54% reduction, respectively) under their experimental conditions and calculation framework. In addition, solvent-free microwave configurations have been described for fresh *C. asiatica* (e.g., 20 g processed in 15 min at 300 W under the reported vacuum/operating conditions), indicating that rapid processing is feasible without added organic solvents in that setup; nevertheless, overall solvent inputs and downstream handling depend on feed moisture and process design [31]. In summary, for *C. asiatica*, the main sustainability levers reported for MAE/UAE/SFME are shorter processing times and lower solvent use, but scale-up performance and environmental benefit should be confirmed case-by-case using robust process metrics.

Consistent with this, *C. asiatica* studies reported that “green” benefits are mainly discussed in terms of shorter extraction time, reduced solvent consumption, and improved selectivity under the stated conditions [53]. Solvent/phase selection also contributes to sustainability: SC-CO_2_ enables solvent removal by depressurization, and enzyme-assisted extraction can improve recovery in milder aqueous or hydroalcoholic systems by reducing extraction severity [22,92]. In practice, industry-aligned systems prioritize low-toxicity solvents (e.g., CO_2_, water, ethanol) to support compliant processing and simplify solvent removal relative to chlorinated or high-toxicity organics [22].

### 4.3. Green Solvents for Bioactive Compound Extraction

Green solvent systems are increasingly coupled with green extraction technologies to recover bioactive compounds from *C. asiatica*, and solvent polarity is a major determinant of extraction selectivity and efficiency [96]. These approaches often employ biodegradable, low-toxicity solvents such as water and ethanol, supporting a shift away from workflows that rely on higher-toxicity organic solvents [96] (Figure 3). Solvent selection and the extraction technique jointly determine which compound classes are preferentially recovered, because both solvent polarity and extraction physics influence solubilization and mass transfer [76].

#### 4.3.1. Solvent Polarity Effects on Bioactive Compounds Recovery

Solvent polarity governs the selective recovery of *C. asiatica* metabolites through differential solubility. Changing solvent ratios can therefore shift extract composition, depending primarily on compound polarity [53]. In microwave-assisted extraction (MAE), increasing ethanol proportion was reported to increase recovery of the triterpenoid glycosides (madecassoside and asiaticoside), while reducing the relative recovery of the corresponding aglycones (madecassic acid and asiatic acid) under the reported operating conditions. This polarity-linked behavior indicates that higher ethanol fractions tend to favor glycoside-enriched profiles, whereas more aqueous conditions tend to favor relatively higher aglycone recovery, although the magnitude of these shifts is method- and protocol-dependent [96].

Methanol is frequently used in laboratory studies because it performs well across a wide polarity range and is often used as a benchmark solvent for method development. However, methanol is not considered a green solvent: its toxicity and regulatory constraints limit its suitability for food, nutraceutical, and many cosmetic manufacturing applications, and it typically necessitates strict solvent-recovery and residue control. For industrially aligned green processing, ethanol–water systems are generally preferred, while NADES and supercritical CO_2_ (often with an ethanol co-solvent for polar targets) are increasingly evaluated as lower-volatility, more compliant solvent platforms for scalable production [76]. The highest concentration of all four compounds was obtained in the ultrasound-assisted methanolic extract, with values of 8.21 ± 0.12 mg/g for madecassoside, 7.82 ± 0.11 mg/g for asiaticoside, 4.44 ± 0.14 mg/g for madecassic acid, and 3.38 ± 0.11 mg/g for asiatic acid [76].

Mechanistically, polarity effects arise from solvent–solute interactions (e.g., hydrogen bonding and dipole interactions) that influence solubility and diffusion in the plant matrix [76]. A 50% ethanol extract of *C. asiatica* has been reported to contain higher levels of total polyphenols and flavonoids, whereas 100% ethanol favored recovery of β-carotene and tannins [36]. Accordingly, the solvent composition reported as ‘optimal’ varies across studies because it depends on the extraction method, operating conditions, and whether the analytical target is glycosides (asiaticoside/madecassoside) or aglycones (asiatic acid/madecassic acid) (Table 3).

Across studies, differences in reported triterpenoid yields reflect both (i) genuine polarity-driven selectivity between glycosides (asiaticoside/madecassoside) and aglycones (asiatic acid/madecassic acid) and (ii) methodological variability among extraction protocols. In particular, comparisons across publications are influenced by differences in solid–liquid ratio, particle size, residence time, temperature, ultrasound/microwave power density, and raw-material variability (genotype/harvest/drying), in addition to solvent composition. Accordingly, ‘optimal’ conditions are interpreted primarily within each study design, while solvent selectivity is used to rationalize the direction of profile shifts (glycoside-rich vs. aglycone-enriched fractions) rather than to rank methods across non-harmonized protocols [53,76,96].

#### 4.3.2. Ethanol-Water Mixture as Green Solvent

Ethanol–water mixtures are widely used as green solvents due to low toxicity, biodegradability, and renewable origin [96]. These solvent systems demonstrate exceptional efficiency in extracting triterpenoid compounds from *C. asiatica*, with different concentrations showing selectivity for specific compound classes [36,96]. The 50% ethanol extract of *C. asiatica* contained a significantly higher number of polyphenols and flavonoids, whereas the 100% ethanol extract contained the highest amount of β-carotene and tannins [36].

Among the concentrations evaluated in [96], 80% ethanol–water provided the highest recovery of triterpenoid glycosides under the reported UAE and MAE conditions (Figure 3). The optimal ultrasound-assisted extraction parameters for *C. asiatica* glycoside extraction were 80% ethanol as the solvent at 48 °C for 50 min [96]. Similarly, microwave-assisted extraction demonstrated optimal conditions using 80% ethanol at 100 watts for 7.5 min, producing the highest triterpenoid content with 7.332 ± 0.386% *w*/*w* madecassoside, 4.560 ± 0.153% *w*/*w* asiaticoside, 0.357 ± 0.013% *w*/*w* madecassic acid, and 0.209 ± 0.025% *w*/*w* asiatic acid [96] (Table 3).

#### 4.3.3. Subcritical Water Extraction (SWE)

Subcritical water extraction (SWE) uses liquid water under elevated temperature and pressure (typically >100 °C, below the critical point) to tune solvent polarity and recover moderately polar constituents. For *C. asiatica*, SWE has been explored as a solvent-minimizing option for extracting phenolics and, under some conditions, triterpenoid markers; however, extraction selectivity and marker recovery are highly dependent on the operating window (temperature–time–pressure) and the analytical endpoint (total extract vs. quantified asiaticoside/madecassoside/asiatic acid/madecassic acid). Because higher temperature and longer residence times can promote hydrolysis or profile shifts—particularly for glycosides—SWE conditions should be optimized according to whether the target is centelloside-rich (glycosides) or aglycone-enriched fractions [102,103,104].

#### 4.3.4. Emerging Green Solvents: Natural Deep Eutectic Solvents (NADES)

Natural deep eutectic solvents (NADES) are low-volatility solvent systems proposed as alternatives to conventional volatile organic solvents for extracting polar phytochemicals. In *C. asiatica*, a NADES system based on acetylcholine chloride–malic acid–water (1:2:2), diluted with water (40:60), produced higher recoveries of the triterpenoid glycosides madecassoside (21.7 mg g^−1^ DW) and asiaticoside (12.7 mg g^−1^ DW) than the >80% (*v*/*v*) ethanol condition reported in the same study (13.3 mg g^−1^ DW MS; 7.80 mg g^−1^ DW AS). Under the reported MAE conditions, NADES extracts also showed higher antioxidant capacity (IC_50_ = 0.26 mg mL^−1^) compared with aqueous ethanol and water extracts. While these results support NADES as a promising solvent platform for glycoside-enriched extracts, scale-up considerations include solvent viscosity, recyclability, and downstream removal/compatibility with drying and formulation requirements [94].

### 4.4. Purification and Standardization

**Figure 4:**
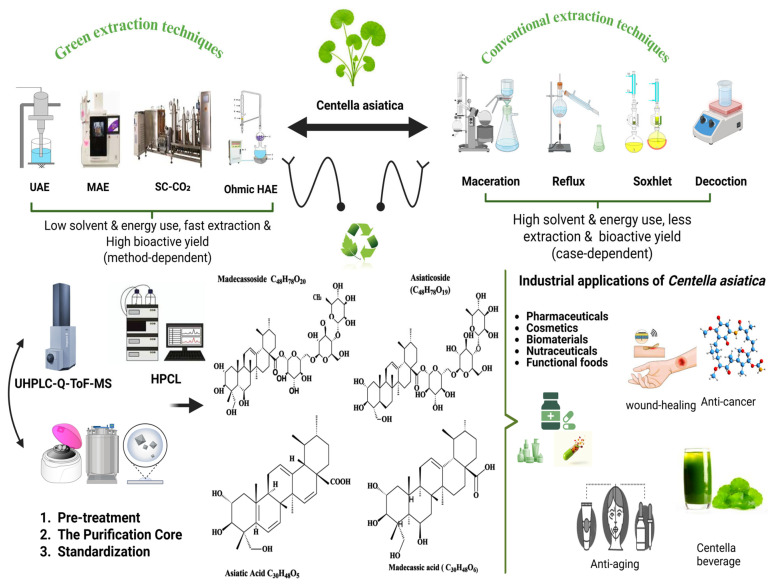
Schematic overview of a *Centella asiatica* biorefinery concept. The figure contrasts conventional extraction routes (maceration, reflux, Soxhlet, decoction) with greener alternatives (UAE, MAE, SC-CO_2_, ohmic heating-assisted extraction) and summarizes a downstream workflow comprising pre-treatment, purification, and standardization, supported by analytical platforms (UHPLC-Q-ToF-MS for fingerprinting/identification and HPLC/UPLC for routine quantification). The final outputs support applications in pharmaceuticals, nutraceuticals, cosmetics, biomaterials, and functional foods.

Purification and standardization are central to producing *C. asiatica* extracts and fractions with reproducible composition and quality suitable for defined applications. In industrially oriented workflows, downstream processing removes co-extractives and enables fractionation into triterpenoid- and polyphenol-enriched streams, which can support multiproduct valorization depending on the process design [88] (Figure 4).

#### 4.4.1. Analytical Platforms for Process Monitoring and Structural Elucidation

Standardization relies on validated analytical methods to monitor key markers, assess batch-to-batch reproducibility, and verify purity through the purification sequence.

Process monitoring (HPLC/UHPLC): HPLC and UHPLC, often coupled with diode-array detection (DAD), are widely used for routine marker quantification and in-process control. These platforms quantify marker triterpenoids, including asiaticoside, madecassoside, asiatic acid, and madecassic acid. For complex extracts, UHPLC–Q-ToF–MS can support broader chemical fingerprinting, detection of minor derivatives, and assessment of profile integrity beyond the main markers.

Structural Identification (NMR): NMR is used for definitive structure confirmation after isolation; it is not a purification technique. In the purification workflow, NMR is employed after isolation for definitive structural elucidation and stereochemical assignments. High-field NMR (e.g., 600–800 MHz) can be useful for distinguishing closely related isomers, such as isomadecassoside, which may exhibit identical mass-to-charge ratios but different structures and potentially different bioactivity profiles [83,88].

A key limitation when comparing quantitative marker levels across studies is analytical non-equivalence. Reported concentrations can differ due to chromatographic conditions (column chemistry, gradient, detection wavelength), reference standards and calibration strategy, sample preparation, and critically, the reporting basis (mg/g dry plant, mg/g dry extract, % *w*/*w* of extract, or enriched fraction). For this reason, quantitative values in this review are treated as method- and matrix-dependent unless studies use comparable validated assays and clearly defined bases. For robust standardization, comparability is strengthened by combining (i) routine marker quantification (HPLC/UHPLC–DAD with external standards) with (ii) orthogonal fingerprinting (e.g., UHPLC–MS) to detect broader profile shifts beyond the four markers [105,106,107].

#### 4.4.2. Sequential Industrial Purification Workflow

Downstream processing from crude extracts to standardized powders typically includes clarification, concentration, fractionation/enrichment, and drying, supported by routine chromatographic quality control. As summarized in (Figure 4), workflows are often described as pre-treatment (clarification/defatting), an enrichment/purification core (e.g., adsorption resins and fractionation), and final standardization, supported by fingerprinting (UHPLC–MS) and routine marker assays (HPLC/UHPLC) [88].

1. Pre-treatment and Defatting: The crude hydroalcoholic extract is typically concentrated. Depending on the matrix and target specifications, an optional defatting step using non-polar solvents (e.g., food-/process-grade hydrocarbons where permitted) may be applied to remove waxes, pigments, and lipids and reduce resin fouling.

2. Enrichment via Macroporous and Ion-Exchange Resins: Macroporous adsorbent resins (e.g., D101, AB-8, HPD100) are commonly used for enrichment of saponin-rich fractions at scale. These resins offer a high adsorption capacity and selectivity for saponins.

Mechanism: In typical operation, triterpenoids are retained on the resin while more polar impurities (e.g., sugars/salts) are washed out, followed by hydroalcoholic elution (increasing ethanol concentration) to recover enriched fractions.

Gradient elution: The target compounds are recovered using a hydroalcoholic elution gradient (e.g., increasing ethanol concentration). This stage can increase triterpenoid enrichment substantially (reported purities vary with resin type, loading, and elution conditions).

3. Polishing and Recrystallization: To reach higher purity specifications, enriched resin fractions may undergo a polishing step. (Figure 4)

Preparative HPLC: Preparative HPLC can be used to isolate individual markers (e.g., asiaticoside) at high purity, although feasibility depends on scale and cost constraints.

Recrystallization: This is the most cost-effective industrial method for final purification of the product. By utilizing specific solvent-temperature profiles, the triterpene markers were crystallized from the mother liquor, with reported final purities commonly in the ~95–98% range under optimized conditions [22].

Chromatographic methods used for *C. asiatica* purification and QC include RP-HPLC/UPLC (commonly C18 phases with acetonitrile–water gradients) for separation and quantification of asiaticoside, madecassoside, asiatic acid, and madecassic acid [108,109]. Depending on the objective, higher-resolution or preparative workflows (e.g., semi-preparative HPLC, HSCCC coupled to HPLC) have been reported for isolating individual triterpenoids or co-occurring flavonoids at high purity, while HPTLC is frequently used for rapid, cost-effective QC in herbal matrices [110,111]. For fingerprinting and traceability, hyphenated MS methods (e.g., UHPLC–MS/MS or UHPLC–Q-ToF–MS) support broader profiling and can be validated to meet linearity/precision/accuracy requirements for targeted analytes in defined matrices [74,112].

Linking extraction conditions to downstream value requires considering chemical profile outcomes, not yield alone. Mild, polarity-tuned extraction (e.g., ethanol–water systems) typically targets glycoside-rich fractions suited to standardized TECA-type preparations, whereas higher thermal stress or extended residence time can shift relative composition (e.g., partial hydrolysis or deglycosylation), thereby changing marker ratios relevant to both quality specifications and functional positioning. Consequently, the workflow presented here emphasizes controlling the upstream extraction window (solvent polarity and process intensity) and verifying profile integrity through marker ratios and fingerprinting before resin enrichment and final polishing, ensuring reproducible composition aligned with intended use (cosmetic, nutraceutical, or phytopharmaceutical contexts) [22,110,111,112].

#### 4.4.3. Biorefinery Integration

Taken together, these approaches support integration of *C. asiatica* processing into the circular bioeconomy and multiproduct biorefinery concepts. The standardized extracts produced through this “Extraction → Resins → Elution → Recrystallization” workflow can provide reproducible profiles suitable for formulation in dermatology/cosmetic and nutraceutical contexts, while therapeutic claims should be interpreted according to evidence level [10,71].

Advancements in extraction and purification methods have significantly enhanced the conversion of *C. asiatica* biomass into value-added products (Table 4). Together, these advances strengthen the plant’s position as a reliable and eco-friendly source of standardized biochemicals for pharmaceutical, nutraceutical, cosmetic, and biomaterial applications.

### 4.5. Integration into a *C. Asiatica* Biorefinery Concept

Green extraction and purification workflows support the separation of *C. asiatica* biomass into complementary biochemical streams [113]. Triterpenoid-rich fractions are suitable for pharmaceutical and dermal formulations, whereas flavonoid and polyphenol-rich fractions can be used in antioxidant and nutraceutical products [88]. Volatile and nutritional fractions derived from secondary streams contribute to applications in aroma, dietary supplements, and biomaterials. Rather than targeting a single ingredient, a biorefinery approach maximizes the total biomass value and aligns *C. asiatica* exploitation with circular bio-economy principles [90,114]. This framework identifies *C. asiatica* as a renewable biochemical feedstock, enabling the simultaneous valorization of multiple metabolite classes through integrated green extraction and purification strategies. (Figure 4) offers a comprehensive overview that contextualizes the concepts discussed in this chapter, comparing conventional and green extraction technologies, detailing the downstream purification of major triterpenoids, and outlining industrial application pathways.

### 4.6. Techno-Economic and Sustainability Considerations

The industrial adoption of *C. asiatica* processing depends not only on extraction efficiency but also on solvent safety, energy demand, scalability, and regulatory acceptance [22]. UAE and MAE currently offer the most favorable techno-economic balance owing to their reduced solvent consumption, shorter processing times, and compatibility with food-grade and cosmetic-grade ethanol [90,114]. SC-CO_2_ enables solvent-free, pharmaceutical-grade extracts and superior thermal protection for triterpenoids, although it requires a higher capital investment and technical expertise [90]. Overall, green extraction coupled with selective purification represents a feasible route for large-scale, standardized biochemical production, minimizing environmental footprints, and supporting the transition to sustainable bioprocessing [115]. The extraction and purification techniques summarized in this chapter enable the reproducible production of standardized biochemical fractions from *C. asiatica*, positioning the plant for high-value industrial applications. These advances directly support the valorization pathways and end-use applications detailed in Section 5.

## 5. Valorization and Industrial Applications of Biochemical Fractions of *C. asiatica*

Biochemical fractions obtained through green extraction and downstream purification (Section 4; Figure 3) enable multiple high-value applications in different sectors. The industrial relevance of *C. asiatica* lies in the valorization of triterpenoid-rich, polyphenol/flavonoid-rich, and volatile fractions, which differ in formulation compatibility, evidence base, and regulatory classification. The main valorization pathways from biomass to sector-specific products are summarized (Figure 4).

### 5.1. Pharmaceutical Applications

*Centella asiatica* extracts and triterpenoid-rich fractions are widely investigated for skin-related applications, particularly wound repair and scar management. The four marker triterpenoids—asiaticoside, madecassoside, asiatic acid, and madecassic acid—have been linked to anti-inflammatory and repair-related effects in experimental models, and selected standardized preparations have been evaluated in humans for specific indications [116]. Mechanistic studies, mainly in vitro and preclinical in vivo, suggest that *C. asiatica* triterpenoids can modulate inflammation-related signaling (e.g., NF-κB and MAPK) and may influence extracellular-matrix remodeling through TGF-β/Smad-related pathways; however, reported mechanisms depend on the specific compound, model, and formulation [27,116,117].

Madecassoside is a key marker triterpenoid and a major bioactive constituent in many standardized *C. asiatica* preparations, and it has been reported to support wound-closure processes and reduce inflammatory markers in preclinical models [118]. In some human studies of madecassoside-enriched topical formulations, improvements in re-epithelialization and collagen-related endpoints have been reported under defined clinical conditions [22].

Standardized *C. asiatica* preparations are marketed in multiple countries, with regulatory classification depending on jurisdiction and intended use (e.g., medicinal product, traditional herbal preparation, or cosmetic ingredient). Examples of marketed preparations reported in the literature include Madecassol^®^, Centellase^®^, and Blastoestimulina^®^, available in topical and oral dosage forms containing titrated extracts of *C. asiatica* (TECA) (Table 5) [22,116]. However, product availability, regulatory status, and stated concentrations may vary by manufacturer, country, and over time and should be verified against current product documentation. Overall, these preparations are used in practice for wound and scar-related indications, with outcomes depending on formulation, dose, and clinical context [116].

### 5.2. Cosmetic Applications

The cosmetic and cosmeceutical use of *C. asiatica* has expanded substantially due to widely reported skin repair and anti-aging-related properties. It is commonly incorporated into serums, creams, and masks, where it is associated with improved skin elasticity and reduced transepidermal water loss in formulation-dependent studies [22]. These effects support its use in anti-aging formulations designed to maintain skin structure and hydration levels.

Triterpenoids, including asiaticoside and madecassoside, have been reported to support fibroblast-related processes and collagen-associated endpoints relevant to dermal remodeling. In addition, antioxidant and photoaging-related effects have been described in experimental systems, supporting its frequent use in daily skincare products designed for long-term skin maintenance [22]. This dual role—supporting repair-related processes and providing antioxidant protection—makes it suitable for daily skincare formulations aimed at long-term skin maintenance. Modern cosmetic brands increasingly include standardized *C. asiatica* extracts under the well-known CICA label in premium skin care lines. Its multifunctional benefits have established it as a widely used ingredient in anti-aging, skin repair, and protective formulations [122].

### 5.3. Nutraceutical and Food Applications

In the nutraceutical sector, *C. asiatica* is marketed globally as a natural cognitive enhancer and an adaptogen [123]. Its stress-modulating and neuroprotective effects support mental clarity, memory retention, and fatigue resistance [22]. Formulations often combine *C. asiatica* with *Ginkgo biloba*, *Bacopa monnieri*, or phosphatidylserine to enhance cognitive performance, particularly in aging populations and professionals exposed to stress [22]. Human studies suggest that supplementation with *C. asiatica* extracts may improve cognitive and anxiety-related endpoints in specific settings; however, outcomes depend on extract type, dose, duration, and study design [22,124].

As a functional food, *C. asiatica* is traditionally consumed across South and Southeast Asia, especially in Sri Lanka, Malaysia, India, and Thailand. Foods such as *Kola Kenda*, *Gotu Kola Sambola*, and *ulam* incorporate the plant for its fiber and glycemic benefits [97]. Modern innovations have extended their use to fortified products such as chocolate, noodles, and beverages [120,125]. Microencapsulated *C. asiatica* extracts have been successfully added to chocolate oat milk, maintaining flavor acceptability while delivering bioactive compounds such as asiaticoside and asiatic acid [97,120]. Its incorporation into yogurt enhances the antioxidant activity and phenolic and flavonoid content and shows antidiabetic potential through α-amylase inhibition [97]. Herbal teas and functional beverages derived from *C. asiatica* also support gut health, probiotic balance, and cognitive alertness [126]. To summarize how distinct biochemical fractions translate into industrial products, Table 5 summarizes sector-specific applications and representative product formats derived from triterpenoid-rich, flavonoid-rich, and volatile fractions of *C. asiatica.*

### 5.4. Industrial Biochemical Valorization and Circular Bioeconomy

*Centella asiatica* biomass can be valorized into multiple sector-specific fractions, with triterpenoid-enriched streams primarily supporting pharmaceutical and dermatological applications, polyphenol/flavonoid-enriched streams aligning with nutraceutical and functional-food products, and volatile fractions contributing to sensory and wellness formulations [22,38,53,127]. When these fractions are co-produced through integrated extraction–fractionation–purification workflows, the overall biomass utilization can be improved and process residues reduced, consistent with circular bioeconomy principles. Rather than treating *C. asiatica* as a single-ingredient source, this approach positions it as a renewable feedstock capable of supplying parallel biochemical value streams to different industrial pipelines [128,129,130].

### 5.5. Emerging Innovation Trends and Commercial Expansion

Recent industrial interest has shifted towards standardized, high-purity biochemical ingredients from *C. asiatica* rather than generic plant extracts [22,131]. Advanced delivery technologies, such as microencapsulation for nutraceuticals and stabilized triterpenoid concentrates for dermatological applications, can improve product stability and support consumer safety and longer-term performance [105]. Market adoption is driven by three converging demands: evidence-based natural ingredients, sustainability of supply, and regulatory acceptance of green extraction technologies such as UAE, MAE, and SC-CO_2_ [22]. Consequently, the global portfolio of *C. asiatica* products is expanding across pharmaceutical, cosmetic, and functional food markets, resulting in rapid commercial growth [22,29]. Collectively, these innovation trends set the stage for evaluating the global market landscape, economic impact, and supply chain challenges presented in Section 6.

## 6. Global Market Landscape and Economic Significance

Commercial interest in *C. asiatica* has increased across cosmetics, nutraceuticals, and (in some jurisdictions) herbal medicinal products, driven by demand for traceable botanical ingredients and standardized triterpenoid markers. More than 100 marketed formulations containing *C. asiatica* extracts have been reported; when stated, marker specifications (e.g., minimum centellosides or minimum levels of individual triterpenoids) are typically manufacturer and product-dependent and should not be interpreted as a harmonized regulatory threshold [36]. In contrast, pharmacopoeial specifications generally refer to quality requirements for the herbal drug or standardized extracts and may define minimum levels for individual markers (e.g., asiaticoside and madecassoside) or for total triterpenoid derivatives depending on the monograph and extract type (e.g., TECA/TTFCA). Commercial interest has also stimulated patent activity, predominantly focused on (i) extraction and standardization processes, (ii) topical formulations and delivery systems (e.g., emulsions, gels, encapsulation), and (iii) combination products positioning Centella-derived ingredients for skin comfort and appearance-related outcomes [36]. Literature sources report a substantial supply–demand gap in Indonesia (reported annual demand ~25 tons versus reported domestic production ~4 tons), with a high reliance on wild collection; however, the cited reports do not always specify whether these figures refer to fresh biomass, dried herb, or extract equivalents, and they are therefore presented here as reported annual values [36,132]. Growing consumer demand for natural and environmentally friendly products is also shaping the pharmaceutical, cosmetic, and nutraceutical sectors, aligning with trends in green extraction and sustainable production [36,133].

### 6.1. Global Market Overview

Market dynamics differ substantially depending on whether the focus is on finished products or ingredient-grade extracts. The *C. asiatica* market spans several sectors, with cosmetics representing a major downstream outlet. However, market assessment is more informative when based on *Centella*-focused segments (e.g., *Centella*-containing skincare products or *C. asiatica* extract ingredients) rather than global cosmetics totals. Available market reports indicate continued growth in Centella-containing skincare and *C. asiatica* extract ingredients, with strong activity in Asia-Pacific and increasing uptake in Europe and North America; however, published estimates vary depending on whether the scope covers finished products or ingredient-grade extracts. Parallel growth exists in nutraceuticals, where *C. asiatica*-based cognitive and mood-support supplements contribute to the USD 300+ billion industry [22]. Standardization plays a central role in scalability, but reported marker thresholds vary by product type and manufacturer and should not be interpreted as universal regulatory requirements [36]. Documented raw-material pricing varies substantially by grade and reporting basis; for example, India has been reported to supply 500–1000 metric tons of dried herb at approximately USD 0.4–0.5 per kg [36,132].

### 6.2. Commercial Innovation, Key Players, and Patent Dynamics

Leading international brands such as La Roche-Posay and Innisfree have integrated *C. asiatica* extracts into premium dermatological products [22,134]. This illustrates commercial uptake of *C. asiatica* ingredients in premium skincare. Across patents, claims are typically framed around composition and formulation performance (e.g., improved stability, delivery, or sensory properties) rather than constituting evidence of clinical efficacy. Patented applications frequently reference madecassoside- and asiaticoside-containing extracts in topical compositions positioned for skin comfort and appearance-related outcomes (e.g., soothing claims, stretch-mark appearance, post-procedure care), but such claims should be distinguished from clinical efficacy unless supported by human evidence [22]. Formulation innovation continues to accelerate, with nanoemulsions developed to enhance dermal penetration and prolong bioactive release [135]. Standardization remains commercially important; however, described marker thresholds vary by product type and intended use, and regulatory expectations differ between cosmetics, supplements, and medicinal/traditional herbal products [36].

### 6.3. Regulatory and Standardization Framework

*C. asiatica* is listed in multiple pharmacopoeias, including Ayurvedic and Chinese pharmacopoeias, supporting its recognized use as a medicinal plant and providing quality references for herbal materials and extracts [7,17,20,25]. However, regulatory expectations vary by product category and route of administration, and global harmonization remains limited.

- Cosmetics (topical): C. asiatica is widely used as a cosmetic ingredient (often marketed as ‘CICA’), where regulatory emphasis is placed on ingredient safety, prohibited substances, and substantiation of cosmetic claims, rather than medicinal efficacy.
- Food supplements (oral): In many markets, C. asiatica products are regulated as supplements, typically requiring safety and quality control while restricting disease-treatment claims [24].
- Herbal medicinal products/traditional herbal products (topical and oral): Where products are classified as medicines or traditional herbal medicinal products, requirements may include more stringent quality specifications, standardized marker control, and product dossiers consistent with pharmacopoeial and GMP expectations [22].

Where specified in monographs and quality references, minimum marker thresholds may include values such as ≥1% asiaticoside and ≥0.5% madecassoside for defined preparations; these should be interpreted as quality specifications linked to the referenced monograph/extract type rather than universal product requirements [7,22].

## 7. Challenges and Future Perspectives in Sustainable Production

The global shift toward sustainable production of *C. asiatica* aligns with rising demand for natural bioactive ingredients in the pharmaceutical, cosmetic, and nutraceutical industries. However, translating laboratory-scale success into industrially viable biochemical production remains challenging. Key challenges include agronomic variability that complicates standardization, limited scalability of some green extraction technologies, and supply chain constraints that hinder long-term competitiveness [22,36]. Overcoming these barriers requires coordinated advancements in plant biotechnology, bioprocess optimization, and value-chain engineering to ensure a reliable, high-quality supply of centellosides.

### 7.1. Agronomic Variability and Extract Standardization

Phytochemical heterogeneity remains a major barrier to commercialization. The concentrations of key triterpenoids in *C. asiatica* accessions show significant variation: asiatic acid (0.00–0.29%), madecassic acid (0.02–0.72%), asiaticoside (0.04–2.41%), and madecassoside (0.15–5.27%) [85]. Geographic origin, soil composition, climate, harvest timing, and post-harvest handling significantly influence this variability, compromising the reproducibility of formulations and their clinical translation [22,136]. Achieving batch-to-batch consistency requires robust quality-control frameworks, including chemotypic markers, validated HPLC chromatographic fingerprinting, and compliance with pharmacopoeial monographs [22]. The full standardization of all four centellosides is increasingly considered necessary for both raw materials and finished products [70]. Transitioning from wild harvesting to controlled cultivation under Good Agricultural and Collection Practices (GACP) is essential to ensure consistent biomass composition and predictable yields [22].

### 7.2. Industrial Scalability of Eco-Extraction Technologies

The transition of green extraction methods from laboratory settings to industrial applications remains a significant challenge [137]. Supercritical CO_2_ extraction (SFE) delivers high selectivity and preserves thermolabile compounds but is limited by its high operational costs and complex infrastructure [22,87]. Ultrasound-assisted extraction (UAE) and microwave-assisted extraction (MAE) reduce solvent use and extraction time but can become less cost-efficient at larger scales [138].

Owing to the varying phytochemical compositions among biomass sources, it is necessary to continually optimize the extraction parameters, such as temperature, pressure, solvent polarity, and pretreatment, for each batch of biomass [119]. Hybrid systems, such as SFE coupled with natural deep eutectic solvents (NADESs) and ionic or bio-based solvents, offer promising sustainability advances but currently face high processing costs and incomplete techno-economic validation [89,139]. Comprehensive environmental and techno-economic assessments (including LCA where appropriate) are needed before industrial adoption can become feasible [140].

### 7.3. Competitiveness and Sustainability Across the Value Chain

The continued use of traditional extraction methods sustains cost-driven competition. Older methods require minimal capital investment and are supported by existing industrial infrastructure, whereas green extraction platforms require higher upfront costs and skilled optimization [137,140]. Biomass variability can further increase production expenses because of the frequent recalibration of extraction parameters [137]. Regulatory requirements related to solvent minimization, waste reduction, and carbon footprint add to operational expenses [140]. To remain competitive, the *C. asiatica* industry must integrate economic sustainability with environmental responsibility. Transparent and traceable supply chains that enable premium pricing for sustainably produced products are crucial. Cooperation across the cultivation, extraction, formulation, and commercial sectors is essential to ensure consistent quality and secure long-term industrial adoption.

### 7.4. Future Perspectives: Biotechnological and Bioprocessing Solutions

Emerging biotechnology offers promising routes for the stable and scalable production of triterpenoids. Biotechnological production systems offer predictable, year-round output, scalability, and simplified downstream processing compared with field cultivation, and easier extraction and purification, compared to traditional plant cultivation [141]. Plant tissue culture systems, such as cell suspension and hairy root cultures, facilitate controlled, contaminant-free, and predictable centelloside production [6,22]. Hairy root cultures have shown transformation efficiencies of 27% in leaves and 12% in petioles, with the potential to yield up to 46.57 mg·g^−1^ of triterpenoids [6,85]. On a molecular level, metabolic engineering and genome editing techniques, including CRISPR–Cas9, are being utilized to enhance key biosynthetic genes and redirect metabolic flux towards high-value triterpenoids [22,142]. The central enzymes in this process include CYP450 monooxygenases, which participate in C6-hydroxylation, and UDP-glucosyltransferases, which are responsible for C28-glycosylation [70]. Hairy root cultures induced by *Agrobacterium rhizogenes* provide a sustainable platform with rapid, hormone-independent growth and genetic stability, making them viable for producing bioactive compounds and plant-specialized metabolites [143]. Advanced biotechnological strategies, including temporary immersion systems, can optimize nutrient uptake and gaseous exchanges. The integration of nanoparticle technology has shown positive effects on phytochemical biosynthesis, suggesting that combining hairy root cultures with innovative elicitors can significantly enrich bioactive compound yields [143,144,145]. The biotechnological frontier also encompasses microbial fermentation platforms that utilize cell factories, such as *Saccharomyces cerevisiae*, and sophisticated plant cell culture systems that provide sustainable sources of complex phytochemicals, independent of climate and geography [146]. These systems benefit from metabolic and genetic engineering tools, including the overexpression of key enzymes and genome-editing technologies, to rationally redesign biosynthetic pathways and overcome natural yield limitations [143,146]. By integrating engineered biomass systems with scalable eco-extraction, it is possible to achieve predictable year-round production of bioactives, supporting long-term market competitiveness, environmental protection, and circular bioeconomy goals [22]. Overall, these scientific, industrial, and regulatory considerations support an integrated ‘field-to-bioprocess-to-market’ framework, enabling *C. asiatica* to transition from a traditional medicinal herb to a reliable biochemical feedstock for pharmaceutical, cosmetic, and nutraceutical value chains.

## 8. Conclusions

*Centella asiatica* is increasingly studied as a source of high-value biochemicals for pharmaceutical, cosmetic, nutraceutical, and functional-ingredient applications. The four triterpenoid markers—asiaticoside, madecassoside, asiatic acid, and madecassic acid—are widely used for quality control and are major contributors to several bioactivities reported for *C. asiatica* extracts; however, other metabolite classes (e.g., phenolics, flavonoids, and volatile constituents) and whole-extract effects may also contribute to observed outcomes. Evidence is strongest for skin-related applications, particularly wound-repair and scar-management endpoints assessed in preclinical studies and, for selected preparations, in human studies. Other proposed benefits, including neuroprotective and cognitive effects, remain less established and are supported mainly by preclinical or emerging evidence. Advances in green extraction and purification can improve process efficiency and reduce solvent and energy use under optimized conditions, but scale-up performance and sustainability benefits should be confirmed using rigorous techno-economic and life-cycle assessments. Future progress will depend on coordinated improvements across cultivation (GACP-aligned production, genotype selection, and standardization), analytical control (validated marker profiling), and scalable processing strategies. Overall, *C. asiatica* represents a promising model biomass for field-to-market development of standardized plant-derived biochemicals when evidence level, quality control, and sustainability claims are carefully aligned with the available data.

## Figures and Tables

**Table 1:** Cultivation variables and quantified changes in triterpenoids of *Centella asiatica*.

Cultivation Variable	Condition(s) Tested	System/Scale	Experimental Context	Plant Age/Stage	Tissue & Sampling Time	AS	MS	AA	MA	Unit/Basis	Analytical Method	References
Light source	LED vs. fluorescent	Hydroponic (DFT)/pilot	PPFD 90–95 µmol m^−2^ s^−1^; 16 h photoperiod; 25/15 °C (day/night); pH 5.6	Mature plants	Leaves; post-harvest sampling	-	-	-	1.25 ± 0.04	mg g^−1^ DW	HPLC	[6,58]
Nitrogen source	NH_4_^+^:NO_3_^−^ ratio = 20:30 (total N = 50 mM)	In vitro (shoot culture)	Liquid MS medium; 35-day culture cycle	Shoot culture	Shoots; day 35	8.9	-	-	-	mg g^−1^ DW	HPLC	[59,60]
Genotype × tissue	Phenotype F (“fringed”)	Greenhouse pot trial	Natural light; 25 ± 2 °C; standard potting mix	Mature plants	Leaves; harvest time not specified	7.9 ± 0.3	9.7 ± 0.6	-	-	mg g^−1^ DW	HPLC	[61,62]
Genotype × tissue	Phenotype F (“fringed”)	Greenhouse pot trial	Natural light; 25 ± 2 °C; standard potting mix	Mature plants	Roots; harvest time not specified	1.2 ± 0.1	3.2 ± 0.2	-	-	mg g^−1^ DW	HPLC	[6,61]
Elicitation	Methyl jasmonate (0.1 mM)	In vitro (hairy root culture)/lab	MS medium; elicitor added at week 3; harvest at week 5	Hairy roots	Roots; week 5	7.12	-	-	-	mg g^−1^ DW	HPLC	[63,64,65,66]
Light intensity	High (300) vs. low (50) µmol m^−2^ s^−1^	Hydroponic (NFT)/lab	PPFD 300 vs. 50 µmol m^−2^ s^−1^; 16 h photoperiod; 25 °C; 4-week treatment	4-week plants	Shoots; week 4	-	-	-	-	N/A	HPLC	[67,68,69]

**Abbreviations:** AS: Asiaticoside; MS: Madecassoside; AA: Asiatic acid; MA: Madecassic acid; DW: Dry Weight; PPFD: Photosynthetic Photon Flux Density; DFT: Deep Flow Technique; NR: Not reported (-).

**Table 2:** Bioactive and nutritional compound classes in *C. asiatica* and their associated biological functions.

Compound Class	Representative Compounds	Reported Content (Range; Unit As Reported) Content (µg/g)	Biological Activities	References
Flavonoids	Quercetin, kaempferol, rutin, catechin, apigenin, naringin	0.72–11.89	Antioxidant, anti-inflammatory, antibacterial, thrombolytic	[80,81]
Phenolic acids	Chlorogenic, caffeic, ellagic, ferulic acids; dicaffeoylquinic acids	–	Antioxidant, neuroprotective, anti-aging	[47,78]
Alkaloids	Ribalinidine, vellarine, hydrocotyline	3.01–3.05	Antimicrobial, bioregulatory, neuroactive	[78,82]
Phytosterols	Campesterol, stigmasterol, β-sitosterol	18.90	Membrane stabilizing, cholesterol-lowering, anti-inflammatory	[80,83]
Tannins/Polyphenols	Proanthocyanidins	4.44–11.96	Astringent, antioxidant, wound-healing	[82,84]
Volatile Terpenes	Caryophyllene, Farnesol, Elemene	–	Antioxidant, antimicrobial, anti-inflammatory	[78,84]

NR: Not reported quantitatively in the cited source (–). Reported concentrations and units are study-specific and may refer to dry plant material or dry extract, depending on the analytical workflow.

**Table 3:** Summary of Green Solvent Systems, Extraction Parameters, and Bioactive Recovery in *C. asiatica*.

Solvent/System	Extraction Method	Optimal Conditions	Compounds Recovered	Yield/Key Findings	References
Water, Ethanol (Green Solvents)	UAE/MAE	Depends on the extraction type	Broad range of bioactives	Non-toxic, biodegradable, renewable; preferred over harmful solvents	[96,97,98]
Solvent Polarity Effects	MAE	Varying ethanol %	Madecassoside, Asiaticoside (glycosides)	Higher ethanol % → Higher glycosides; lower ethanol % → Higher aglycones	[96,99]
50% Ethanol	General extraction	–	Polyphenols, Flavonoids	Highest polyphenols + flavonoids	[36,97]
100% Ethanol	General extraction	–	β-Carotene, Tannins	Highest β-carotene + tannins	[36,100]
80% Ethanol (Reported optimum)	UAE	48 °C, 50 min	Glycosides	Highest triterpenoid glycoside yield	[96]
	MAE	100 W, 7.5 min	Glycosides + Aglycones	7.332% MS, 4.560% AS, 0.357% MA, 0.209% AA	[96]
Energy Savings (Green methods)	UAE/MAE	Compared with maceration	–	UAE saves 54%, MAE saves 59% energy	[53,96]
NADES (Acetylcholine chloride: malic acid: water)	MAE + NADES	Ratio 1:2:2; Water 40:60	Madecassoside, Asiaticoside	21.7 mg/g MS, 12.7 mg/g AS (≈60% higher than ethanol systems)	[94,97]
NADES advantages	–	–	–	High antioxidant activity (IC_50_ = 0.26 mg/mL); free of conventional volatile organic solvents (VOCs), low volatility solvent system	[94,101]

**Table 4:** A comparison of conventional and green extraction techniques for *C. asiatica*, summarizing solvent systems, extraction conditions, triterpenoid yields, and main advantages and limitations. The data indicate that ultrasound and microwave-assisted extractions often achieve among the highest triterpenoid recovery with reduced solvent use and processing time, supporting their potential as core unit operations in sustainable biochemical production chains.

Extraction Method	Solvent(s) Used	Extraction Conditions	Reported Yield	Advantages	Limitations	References
Maceration/Reflux	Ethanol–water (70–95%)	25–70 °C, 12–24 h	Asiaticoside ≈ 18.2 mg/g DW; madecassoside ≈ 9.5 mg/g DW	Simple, inexpensive, widely available equipment	Long duration, high solvent use, poor selectivity, degradation risk	[36,88]
Soxhlet	Ethanol or methanol	60–80 °C, 6–8 h (continuous reflux)	Total triterpenes ≈ 30.9 mg/g DW	Exhaustive, reproducible	High temperature, solvent removal/drying required; residual levels are process-dependent, not environmentally friendly	[53]
UAE	Ethanol–water (reported: 75% EtOH)	87.5 W, 30 °C, 30 min	Asiaticoside 37.56 ± 4.25 mg/g; Madecassoside 16.91 ± 1.28 mg/g (basis as reported)	High yield, mild temperature, short time, low solvent	Requires equipment, optimization per matrix	[76,97]
MAE	Ethanol–water (reported: 70% EtOH)	500 W, 80 °C, 15 min	Total triterpenes ≈ 81.3 mg/g DW	Rapid, efficient, reduced solvent	Risk of overheating, limited scalability	[53]
SWE	Water	100–180 °C, 10–30 min, 10–15 bar	Rich in polar phenolics; low triterpenoid yield	Green solvent, selective for hydrophilic compounds	Degradation of glycosides at high T/P	[53]
SFE	CO_2_ + 5–10% EtOH (co-solvent)	35–60 °C, 150–300 bar, 60 min	High-purity triterpenes and saponins; yield ≈ 70–85 mg/g extract	Low residual-solvent concern after depressurization; selective; protects thermolabile compounds	High capital cost, complex operation	[22,87]
OHAE	Ethanol–water (60–80%)	60–80 °C, 10–20 min (electric current heating)	Comparable total triterpenes to UAE (≈80 mg/g DW)	Uniform heating, fast, low solvent	Limited data for *C. asiatica* requires scale-up validation	[22]

Reported yields are reproduced as provided in the original studies. Values may be expressed on different bases (e.g., mg/g dry plant material (DW), mg/g dry extract, or % *w*/*w*). Basis is indicated within each entry where available.

**Table 5:** Industrial applications of biochemical fractions of *C. asiatica in* the pharmaceutical, cosmetic, nutraceutical, and biomaterial sectors.

Biochemical Fraction	Sector	Application	Example Formulations (Reported Examples)	Evidence Level	References
Triterpenoids (AS, MS, AA, MA)	Pharmaceuticals	Wound-repair support; scar management; inflammation-related endpoints	TECA-type extracts; reported marketed products (e.g., Madecassol^®^)	Human + preclinical (indication-specific)	[11,22]
Triterpenoids	Dermatology/cosmetics	Skin barrier support; appearance-related anti-aging claims; photoaging-related endpoints	“CICA” creams/serums; liposomal gels; patches	Mainly cosmetic/human use + preclinical	[119]
Flavonoids & polyphenols	Nutraceuticals	Antioxidant-related claims; stress/cognitive-support positioning	Capsules; blends with other botanicals	Mixed human/preclinical; formulation-dependent	[22,120]
Flavonoids & polyphenols	Functional foods	Antioxidant enrichment; phenolic fortification	Fortified beverages/yogurt/chocolate/noodles	Food studies + in vitro; product-dependent	[97,118]
Volatile/aromatic fraction	Wellness/aroma	Sensory-related uses (aroma/flavor)	Herbal teas; beverages; aroma blends	Traditional/marketed use	[121]
Triterpenoids + polyphenols	Advanced delivery	Controlled release/enhanced delivery (formulation claims)	Nanoemulsions; phytosomes; hydrogels; nanofibers	Mostly preclinical	[120,121]
Purified triterpenoids	Biomaterials/tissue engineering	Biomaterial functionalization for repair-related endpoints	Scaffolds; polymer films	Preclinical	[7,38,53]

## Data Availability

No new data were created or analyzed in this study. Data sharing is not applicable to this article.

## Abbreviations

AA	Asiatic acid
AS	Asiaticoside
BDNF	Brain-derived neurotrophic factor
*C. asiatica*	*C. asiatica*
CAGR	Compound annual growth rate
CEA	Controlled-environment agriculture
CICA	*C. asiatica*–based standardized extract
CRISPR–Cas9	Clustered regularly interspaced short palindromic repeats/CRISPR-associated protein 9
CYP450	Cytochrome P450 monooxygenases
DAD	Diode-array detector
DW	Dry weight
EtOH	Ethanol
ECM	Extracellular matrix
FGF	Fibroblast growth factor
GACP	Good Agricultural and Collection Practices
GC–MS	Gas chromatography–mass spectrometry
GMP	Good Manufacturing Practice
HPLC	High-performance liquid chromatography
IC50	Half-maximal inhibitory concentration
ISO	International Organization for Standardization
LC–MS/MS	Liquid chromatography–tandem mass spectrometry
MA	Madecassic acid
MAE	Microwave-assisted extraction
MAPK	Mitogen-activated protein kinase
MEP	Methylerythritol phosphate pathway
MS	Madecassoside
MVA	Mevalonate pathway
NADES	Natural deep eutectic solvents
NF-κB	Nuclear factor kappa-light-chain-enhancer of activated B cells
NMR	Nuclear magnetic resonance
OHAE	Ohmic-heating extraction
PGPR	Plant growth-promoting rhizobacteria
RP-HPLC-DAD	Reverse-phase HPLC with diode-array detection
SC	Supply chain
SC-CO_2_	Supercritical carbon dioxide
SFE	Supercritical fluid extraction
SWE	Subcritical water extraction
STAT	Signal transducer and activator of transcription
TIS	Temporary immersion system
TECA	Titrated extract of *C. asiatica*
UGTs	UDP-glycosyltransferases
UAE	Ultrasound-assisted extraction
UPLC	Ultra-performance liquid chromatography
VEGF	Vascular endothelial growth factor
